# On the Motions and Sounds of the Heart

**Published:** 1834-01-01

**Authors:** 


APPENDIX.—*On the Motions and Sounds of the Heart.*

"In the section on the Auscultation of the Heart, it was stated, that the subject had given rise to many conflicting opinions; and as it was judged inexpedient to confuse the mind of the student in auscultation by a multiplicity of views, the best established and most important points were alone exposed, and all doubtful explanations and applications were avoided. It is of considerable utility in the examination of a controverted point, to review fairly the various opinions respecting it, and by collating them with available facts, to determine the comparative probability of these views: if this had been done with regard to the present subject, much useless speculation might have been saved, and some animal life spared; for any attentive reader of the periodic medical literature, must have perceived that the same opinions have been broached, refuted, and revived by successive writers, and the same experiments performed and reiterated in apparent ignorance of preceding inquiries.

On this account, and to justify what has been set forth in the text, I am induced to give a summary sketch of the leading features in the views which have been advanced respecting the motions and sounds of the heart, and bring them successively to the test of some well-established pathological or physiological facts. Others, besides the names quoted may have supported the views in question, but it is only the views which I wish to deal with, and I cite the writers with a wish to show that the arguments which each has advanced have been carefully studied.

1. M. Laennec. *a.* 1st sound, impulse, and pulse, caused by the ventricular systole. *b.* 2nd sound by the systole of the auricles.—*Remarks. a.* Generally admitted, and proved by various facts and experiments. *b.* Disproved by the fact noticed by Harvey and Haller, and confirmed by modern experiments, that the auricular contraction immediately precedes that of the ventricles; also by this fact, that both sounds sometimes continue after the auricles have ceased to contract.*

2. Mr. Turner.† 2nd sound produced by the falling back of the heart on the pericar-

* Dr. Hope's Experiments on Asses. See his Work, p. 36.

† Med. Chir. Trans. Edin. Vol. III.

dium after the systole of the ventricles.—*Remark.* Disproved by the fact that the sound continues when the heart pulsates out of the pericardium.

3. Dr. Corrigan.* *a.* Impulse and 1st sound caused by the rush of blood into the ventricles during the auricular systole. *b.* 2nd sound by the ventricular systole, which he considers to be instantaneous.—*Remarks. a.* Disproved by the clearly ascertained facts that the 1st sound and impulse accompany the systole of the ventricles when the auricles have ceased to contract. *b.* Disproved clearly in large animals by the ventricular systole, (which is not instantaneous) and the pulse of arteries near the heart, evidently preceding the 2nd sound;† and further disproved by several pathological phenomena.

4. Dr. David Williams.‡ 2nd sound caused by the flapping open of the auriculo-ventricular valves against the sides of the ventricles; these valves he supposes to be opened by the musculi papillares.—*Remark.* This is contrary to the received opinion of anatomists with respect to the functions of the auricular valves and musculi papillares, and there is no collateral argument to maintain so gratuitous an assumption.

5. M. Pigeaux.§ *a.* 1st sound produced by the blood rushing into the ventricles at the moment of their diastole. *b.* 2nd sound by the collision of the blood against the walls of the aorta and pulmonary artery. *c.* The ventricles contract in a moment of silence before the second sound. *d.* The intensity of the sounds proportioned to the force by which the blood is impelled.—*Remarks. a.* Opposed by the facts stated against 3 *a;* opposed also by many pathological facts, such as the occurrence of a murmur with the 1st sound in case of diseased semi-lunar valves. *b.* Disproved by the fact that the 2nd sound occurs distinctly *after* the pulse in the carotids, and therefore after that in the larger arteries. *c.* Opposed by the observation that the 1st sound and ventricular systole occur together and correspond in duration. *d.* This is opposed by the morbid phenomena of dilatation of the ventricles, which always increases the first sound, and of hypertrophy, which diminishes both sounds.

6. M. Majendie.|| 1st sound and impulse produced by the ventricular diastole impelling the apex, the 2nd sound by the systole impelling the base of the heart against the walls of the chest.—*Remark.* Disproved by the facts opposed to 2, and 5, *b.*

The following opinions require a fuller discussion.

7. M. Rouanet.¶ *a.* 1st sound caused by the closing of the mitral and tricuspid valves against the auriculo-ventricular orifices during the ventricular systole. *b.* 2nd sound by the reaction of the blood in the arteries on the semilunar valves at the moment of the ventricular diastole.

8. Mr. H. Carlile.** *a,* 1st sound produced by the rush of blood into the arteries during the ventricular systole. *b.* 2nd sound by the reaction on the semilunar valves as stated in *b* 7.

9. Dr. Hope. *a.* 1st sound and impulse, caused by the ventricular systole. *b.* 2nd sound and *back stroke,* or 2nd impulse, by the ventricular diastole. The natural as well as morbid sounds produced by the motions of the contained fluid.

Before we sift the questionable points in these three last views, it will be proper to review the principal grounds on which we adopt their description of the sounds and motions, in defiance of many preceding authorities. Having been present at some of Dr. Hope's experiments on the ass, I had ample opportunity of convincing myself that the sounds were connected with the motions of the ventricles only. When the pericardium was laid open, and the large heart exposed, vigorously pulsating; the eye watching it, the hand grasping it, and the stethoscope applied to it, gave perfectly corresponding impressions, insomuch, that on substituting touch for hearing, it was difficult to banish the impression that one still *heard* the double sound which was so exactly represented in quality and duration by the motions of the ventricles, as felt and seen; and on combining touch and hearing, by applying the hand and the stethoscope at the same time, these

---

* Trans. of King's and Queen's Coll. of Phys. Ireland.

† Dr. Hope's Experiments, p. 31 of his work; and those of Mr. Carlile, Dublin Journal of Medic. Sci. Vol. IV.

‡ Edin. Med. and Surg. Journ. Oct. 1829.

§ Arch. Génerales de Médecine, Juillet et Novembre, 1832.

|| In a Lecture read at the College of France, quoted by M. Pigeaux.

¶ Journ. Hebdom. No. 97; also Mr. Bryan, Lancet, Sept. 1833.

** Dublin Journal of Medical Science, Vol. IV. The essay was likewise read at the Cambridge Meeting of the British Association.

impressions, which corresponded in nature and duration, were found also to be perfectly simultaneous. The apex of the heart was observed and felt to strike against the ribs at each systole, and thus was explained the impulse. The motions of the auricles, when regular, preceded the ventricular motions and sounds; they were slight and undulatory, increasing from the sinus to the appendix, where they terminated suddenly, and were immediately followed by the ventricular systole. They afterwards became irregular, sometimes failing and sometimes occurring twice slightly during the period of ventricular repose, and in one experiment, entirely ceased some minutes before the movements and sounds of the ventricles. In no instance were they attended with any perceptible sound. This account is confirmed by the experiments of Mr. Carlile, which satisfactorily explain the succession of the motions of the auricles and ventricles; but they were performed on animals too small to illustrate the sounds. He very justly shews that the pulse cannot be simultaneous in all the arteries at once, but must be successive, transmitted in a wave from the heart to the end of these elastic tubes.

Although it seems fairly established that the first, or dull sound, is produced by the systole of the ventricles; and the second, or quick one, by their diastole, it is by no means clearly explained in what way these actions generate these sounds. The following causes have been severally assigned as physically capable of generating the first sounds during the systole of the ventricles. 1. The collision of the particles of fluid in the ventricles. (Dr. Hope.)—2. The rush of blood into the great arteries. (Mr. Carlile.)—3. The closing of the mitral and tricuspid valves. (M. Rouanet, Mr. Bryan.)—4. The muscular contraction itself.

The first of these explanations is ingeniously proposed by Dr. Hope, but he advances no facts in direct proof of the hypothesis. In a number of experiments which I have made on the generation of sound, I have found liquids of all bodies, the most difficult to excite to sonorous vibration; and although they readily transmit vibrations already produced in solids, it requires a combination of circumstances to make them originate sound. This is consistent with the explanation which I have given of the production of sound, (p. 6, *et seq.*) for impulses which throw solids into sonorous vibrations, are expended in liquids in causing a displacement of their particles. On making an experiment with a gum-elastic bottle, by filling it with water, and then forcibly compressing it under water by the end of the stethoscope, (avoiding the use of the hand, for that produces its own muscular sound) I have failed in procuring any sound at all approaching to that of the heart's contraction. The blood yields readily to the contracting ventricle, and there being no obstacle to the escape of blood from it, further than the weight of the arterial column, which the normal action of the heart can quietly and steadily overcome, it passes into the arteries without vibration. But if there be an obstacle to the current of the blood from the ventricle, whether that obstacle be a narrowing or a projection in the orifice, the current will act on it just as the bow does on the string of a violin; a sound will be excited, and thus are produced valvular murmurs. Again, if instead of the orifices being narrowed, the heart contracts with unnatural briskness, expelling its contents with convulsive energy, the natural outlets then become relatively narrow, and are thrown into vibrations; this is the *rationale* of the bellows murmur which accompanies the jerking pulse of pericarditis and the irritation of inanition. But the difference of these sounds, and of the circumstances that excite them, from those of the normal action of the heart, makes me hesitate to refer the latter to the same principle; and the fact that the morbid are often superadded to the natural sounds, also inclines me to think that they have a distinct cause.

2. The second explanation of the first sound, the rush of blood into the larger arteries, is perhaps less liable to the acoustic objection before urged, than the preceding opinion, for the blood has acquired an impulse when it enters the arteries, and if its course there is not free, it might readily produce a sound. But in their natural state, the arteries give passage to the blood as smoothly as the heart parts with it, and it would prove an imperfection in nature were it otherwise. Moreover, if this explanation were true, the large arteries rather than the heart would be the principal seat of the sound; and the sound should be increased by an hypertrophied heart, with a strong pulse, and diminished by a dilated heart and a weak pulse; yet the reverse of these is presented in nature.

3. The closing of the auricular valves. The principal objection to this as the only cause of the first sound, is, that it must be instantaneous, and confined to the first part of the ventricular systole, whereas we know that the first sound is prolonged during the whole period of this action.

4. Although Laennec referred the first sound to the systole of the ventricles, he did not attempt to define the physical cause of its production. In the former edition of this work, I ventured to class it among the muscular sounds which Dr. Wollaston* first noticed to occur in all cases of rapid muscular contraction. This sound may be exemplified by applying the fleshy part of the thumb to the stethoscope or naked ear, and bending and straightening the thumb. It is louder in muscles that are thin, and in a state of considerable tension; and it is remarkable that it does not cease with the apparent movement, but continues as long as the muscle remains contracted and tense: it then takes on an intermitting character like the noise of the rolling of a carriage over rough pavement, whence Dr. Wollaston was led to infer that muscular action is not perfectly continued, but consists of a series of minute contractions and relaxations. A good example of it may be obtained on applying the stethoscope to the neck of a person who holds his head back towards the opposite side, and then throws the platysma myoides into contraction. It still appears to me, that the most simple and satisfactory way of accounting for the first or systolic sound of the heart, is to refer it to this class of sounds. Their physical production seems to depend on the tension into which the fibres of muscles are thrown when they contract; and the self-acting power of these fibres constitutes them the motors as well as the subjects of sonorous vibrations. Here we have to remark the extreme facility with which the motions of solids produce sounds, compared with those of fluids; for it is almost impossible to touch, stretch, bend or compress solids, without throwing them into sonorous vibrations. The varieties observed in the contraction of the heart seem to me to be perfectly explicable on this principle. The sound begins the moment the fibres arrive at a state of tension; it continues until the contraction is completed and the blood expelled from the ventricle, and ceases the instant of the diastole. To perceive more readily the effect of hypertrophy, and of dilatation, let us attend to the sounds produced by the tension of linen or canvass, (for muscle is, mechanically speaking, equally a web of fibres,) and we shall find that in proportion as we thicken the substance, we obscure the sound which is produced on briskly stretching it; but when we use thin and simple webs, the sound becomes proportionally loud and clear. I shall not pursue the illustration of this explanation further, for I introduce it here only interrogatively, as deserving a place among other views, on the claims of which, future observation and experiment must decide. I must only remark, that M. Pigeaux is in error when he maintains that muscular sounds cannot be produced under water: I find them more distinct and free from adventitious sounds of the surface, and I have been able to imitate the sounds of the heart very exactly by muscular movements of the hand under water. I will conclude with the question, if the sounds of the heart are produced by another cause, what becomes of the muscular sound in this case of rapid muscular contraction?

We now come to the subject of the second sound, which, although certainly occurring at the moment of the diastole of the ventricles, has received several different explanations as to its physical cause. The only two which appear tenable in the present state of our knowledge are—1. The reaction of the arterial columns of blood against the semilunar valves. 2. The impulse of the blood from the auricles refilling the ventricle at its diastole.

1. The first of these bears a very inviting aspect, for the 2nd sound is just of that abrupt flapping character that might be supposed to result from the action of a thin valve. But it may be objected to this view, that the arteries more than the heart, should be the seat of this sound. The tense column which throws these valves into play, should receive their shock more forcibly than the heart, which at that moment has become flaccid, and ill adapted to transmit sound or impulse (backstroke) through the whole of its substance. There are some cases of disease which seem also to militate against it. In a case described by Dr. Hope, the 2nd sound on the left side was quite distinct, yet the aortic valves were found in a state of complete rigidity. (Case 20). In another case, the 2nd sound was remarkably loud on the left side, with a weak pulse; yet, after death there was found disease of the mitral valve permitting free regurgitation, and contraction of the aorta: this combination of disease, must have diminished the action of the aortic valves. (Case 15). The action of these valves will be strong, in proportion as the arteries are well filled, and the pulse strong, and the 2nd sound should in this view be proportionally loud. On consulting the records of some cases of this description, I have not found this correspondence. Still I do not consider this view entirely disproved, and it should claim attention in future investigations.

---

* Croonian Lecture, Phil. Trans. 1810.

2. This is Dr. Hope's explanation of the second sound: when the diastole takes place, the blood impelled by a number of concurrent circumstances, shoots with instantaneous velocity from the auricles into the ventricles; and the reaction of the ventricular walls on its particles, when their course is abruptly arrested by the completion of the diastole, is, he conceives, the cause of the loud, brief, and clear sound. The concurrent circumstances which impel the blood into the ventricles at the moment of their diastole, are the distention of the auricles in which the blood has been accumulating during the ventricular contraction; the weight of the ventricles collapsing on the auricles thus distended; the width of the auriculo-ventricular orifices; and lastly, the sucking power of the ventricle in its diastole. With respect to this last, Dr. Hope does not assume that the ventricles have an actively dilating power further than what proceeds from the physical elasticity of their parietes, but such a power has been ascribed to them by Bichat, Pechlin, Carson, and others, and even by Laennec; and although opposed to what we at present know of animal dynamics, it would be rash to absolutely deny the possibility of its existence. The injection of the coronary arteries, which occurs the instant the systolic action ceases, may somewhat contribute to the dilatation of the ventricles. Whatever be the cause, the diastole in large animals is sufficient to force open the hand of a person grasping the ventricles, and it is therefore not surprising that this should have been ascribed to an actively dilating power. It is in favour of Dr. Hope's explanation of the second sound, that it does not falsify Laennec's signs of disease of the auricular valves; and although for acoustic reasons before stated, I should be inclined to place the seat of the sound in the parietes of the ventricles, rendered momentarily tense by the sudden influx of blood, rather than in the motions of the fluid, I incline to this explanation of the cause of the second sound. It needs, however, as Dr. Copland observes, further confirmation: I would add, that the whole subject of the sounds of the heart requires further research; and I shall have accomplished a good object, if these remarks should induce Dr. Hope, who has already thrown so much light on it, to follow up the investigation, until he shall have cleared away the difficulties and doubts that at present beset it."

To those who have not the first edition of Dr. Williams' work, we strongly recommend the present one. To those who hold it, we have afforded an advantage of some consequence. The Appendix is deserving of the consideration of all our readers.

